# Does the psychological profile influence the position of promising young futsal players?

**DOI:** 10.1371/journal.pone.0224326

**Published:** 2019-11-12

**Authors:** Leandro Álvarez-Kurogi, Wanesa Onetti, José Carlos Fernández-García, Alfonso Castillo-Rodríguez

**Affiliations:** 1 UNIR, International University of La Rioja, Faculty of Education, Logroño, Spain; 2 Department of the Languages, Arts and Sport Didactic, Universidad de Malaga, Andalucía-Tech, IBIMA, Malaga, Spain; 3 Department of Physical Education and Sports, University of Granada, Granada, Spain; La Inmaculada Teacher Training Centre (University of Granada), SPAIN

## Abstract

Stress control as well as other psychological characteristics influence sports performance (SP) and could be relevant according to the playing position in team sports, such as the futsal where players have different specific functions within the team. The aim of this study was to analyze the psychological characteristics and profile related to SP of top-level young futsal players, according to the offensive or defensive role. A total of one hundred sixty-seven young promises futsal players participated in this study (84 U16 and 83 U19) and have been chosen to play Championship of Spain Selections. The Psychological Characteristics related to SP for soccer players Questionnaire was used, and one-way ANOVA test was performed based on the playing position (goalkeeper, defender and defender-wing, wing and wing-defender, pivot and wing-pivot, and universal). Results showed that goalkeepers had the best psychological profile and characteristics related to SP. Pivots and wing-pivots had less self-confidence, and universals players, less stress control in relation to the rest of the playing positions (*p* < 0.05). The main findings revealed that the psychological characteristics and profile related to SP in young promises futsal players are different according to the playing position, and this study suggest the inclusion of psychological-training programs in order to improve the psychological abilities of players, especially for players with offensive role who seek to score goals.

## Introduction

Futsal is a sport that is known and practiced worldwide. High-intensity but short-term actions are developed with constant accelerations and decelerations, with a short recovery time between efforts [1]. For this reason, as in the sport of basketball or handball, unlimited changes can be made between players during an official match. In this way, it is interesting to know and control all situations and characteristics of players that can cause both an increase and decrease their sports performance (SP), due to factors such as biomechanical, physical, physiological, nutritional, and psychological, among others [2]. For these reasons, many researchers have as an interest focus to study the futsal sport to encourage and expand the reliable information related to SP to coaches and professionals [3].

One of the essential elements of futsal and other team sports is their psychological aspect, joined with the technical-tactical and physical fitness aspects. Certain psychological aptitudes are necessary for a high-level player [4], given that this aptitude will exert either a positive or a negative influence on the technical-tactical and physical elements of the game [5]. Unfortunately, studies of futsal in relationship to these psychological aspects are recent and focus on only one or scarce psychological aptitudes, and do not present a complete profile of the samples analyzed [1, 6–13]. However, the study of do Nascimento et al. [14] identified the psychological profiles of professional Brazilian soccer players both in males and females, and their influence on the physical aptitude. They concluded that certain psychological profiles (e.g. self-centeredness, boldness, sensuality, insecurity, sensitivity, integrity, and emotionalism) could help players to improve their SP in specific functions like the impulsivity of the futsal player that there is variance according to the offensive and defensive role [15] or resolve problematic situations of the game, and that, there may be different responses to the same situation.

On the other hand, in regard to psychological abilities, e.g. stress control [6, 9, 10] and decision making [8, 11, 12], in futsal have been studied more specifically, taking into account that control of anxiety, self-confidence, motivation, and concentration improve SP, and in the soccer sport, these characteristics are related to the development of the talent of the soccer player [16]. Stress symptoms have been analyzed in professional futsal players, and the findings showed that these symptoms could be increased when repeated sprints tasks are included in preseason training sessions, not only do sprint times improve, but similar improvements could be seen in general performance and stress-reduction [10]. These improvements were evident, since self-confidence and attitude could contribute to the stress control and anxiety [17]. There exists an inverse relationship between self-confidence and anxiety in soccer [18, 19], handball [20] and futsal players [21], and other specific situation as the sports injury [22]. Weinberg and Gould [23], as well as Lorenzo et al. [24], found that this stress control could be improved by the player experience.

Other characteristics, e.g., mental attitude and preparation, are also considered important in this sport, but no scientific information has been published previously on this topic. For mental preparation, López-López et al. [25] suggested the use of psychological techniques on soccer players which foster an effective attitude, e.g., positive self-talk, objective analysis, and imagination. On the other hand, concentration is considered to be a learned ability that can be improved with practice [26, 27], and De la Vega [28] recommended that players should begin to acquire it at an early age. This concentration could be related with the role of the player [29] with more judicious or prudent decisions in players with a defensive role. The defensive behavior, mainly aimed at recovering the ball [30], requires a high level of concentration [29]. These conditions lead us to hypothesize that the performance of attacking and defensive functions could be marked by cognitive and emotionally differentiated abilities, some of which may be associated with inherent personality traits of the athlete [31].

In regard to the playing positions, studies in handball [4, 20] and in soccer [32] showed that the goalkeeper position has the best psychological characteristics prior to the competition. These team sports are related to the futsal, since soccer has similar technical tactical characteristics, and handball has similar regulatory, spatial and behavioral characteristics. Consequently, the development of these psychological characteristics acquires special priority in order to improve the evolution of future high-level player [33]. In elite futsal sport, goalkeeper is the most important playing position during the competition and is different from the other team members [34], because its performance to a great extent determines the success or the failure of the team. This specificity comes marked by its positioning in the field (last player to defend the goal) and for being the only player who has a space of the delimited field where its behavior can be different with respect to the other players [35]. Therefore, the goalkeeper represents a position of responsibility and is trained for this purpose. With respect to studies related to psychosocial and behavioral variables, in other playing positions such as pivots or players with offensive roles, have not been found. In this sense, the starting hypothesis that we propose in this study is that the goalkeepers have better psychological characteristics prior to the competition and therefore, a better psychological profile than other playing positions; and on the other hand, the concentration is higher on player with a defensive role.

Nevertheless it indicated above, studies of futsal still appear to be insufficient [3, 36, 37]. This is evidenced by the lack of research in the first systematic review of futsal has only recently been published [3]. In addition, Palluci et al. [37] emphasize the need to develop applied methodologies in order to perform standardized analysis. Most of the studies published are related with high-level players [3], but it is important to build on this knowledge in order to provide better and more adequate information to improve the training sessions, and consequently the SP of the players [36, 37]. In the same way, the aim of the present study is to identify the psychological characteristics related to SP and the psychological profile of young promises futsal players according to the playing position.

## Materials and methods

### Participants

One hundred sixty-seven young futsal players from 14 years old to 19 years old were the voluntary participants in the actual study, 84 players under 16 years of age (U16; *M* = 59.6 kg; *SD* = 1.5 m) and 83 players under 19 years of age (U19; *M* = 69.7 kg; *SD* = 1.8 m). All were candidates for the Spanish National Futsal Team and belonged to a federated club, competing at the highest level in the U16 and U19 categories. All the players live in the same Autonomous Community from Spain within 100 kilometers of capitol where the team training takes place. They signed a voluntary consent form in compliance with the guidelines found in the Helsinki Declaration (2013) which establishes ethical principles for investigations using human beings. In the case of under-aged players, their parent or guardian signed. The study was approved by the Ethics Committee of the University of Granada (471/CEIH/2018).

The inclusion criteria were to be a member of a federated club and have been chosen to be part of the Spanish National futsal team. Also the player should play in weekly matches with his respective club. The exclusion criteria were being injured at the time of the psychological assessment, to have been injured three months before the psychological assessment, to be penalized by the Sanctioning Committee for a previous expulsion in an official competition match, and players who failed to answer three or more questions of the Psychological Characteristics related to SP test. Firstly, two hundred participants were selected, although thirty-three players were excluded. Twenty-three questionnaires were incomplete, with at least three questions unanswered, and ten players were excluded too, because they had a bone, articular or tendon injury 3 months before the tests. These injuries caused them not to play weekly with their teams and the trainings could have been less intense than those developed by their colleagues. So, the final sample was composed of 167 young futsal players.

### Measures

#### Physical and socio-demographic characteristics

The participants completed an ad hoc questionnaire with questions about their physical person (height, weight, age), and their social-demographic characteristics as a futsal player (category, playing position, years being federated, whether or not family members attended the match). The independent variables were category (U16 or U19); playing positions, reduced to five elements, using a modification of an initial classification done by Santana [38]: goalkeepers, defenders and defenders-wings, wings and wings-defenders, pivots and wing-pivots, and universals; and the experience, measured by years being federated, according to the classification used by Ericsson [39] and Olivier et al. [40].

#### Psychological characteristics related to sports performance

Secondly, the participants completed the Psychological Characteristics related to SP for soccer players Questionnaire [25] in order to assess the psychological profile. This questionnaire is the Spanish translation, adapted and validated, of the Psychological Skills Inventory for Sports [41]. It consists of 40 items with responses given on a five-point Likert rating scale: *strongly agree* (4), *agree* (3), *neutral* (2), *disagree* (1), *strongly disagree* (0), and a sixth option, *« I don't understand »*. The questionnaire is divided into 5 scales or factors: self-confidence (items 5, 13, 15, 17, 27, 33, 36, 37, 39), mental attitude and preparation (items 3, 14, 16, 18, 25, 26, 28, 31), stress control and anxiety (items 2, 6, 7, 9, 11, 12, 19, 23, 24, 30, 38), concentration (items 1, 4, 10, 22, 29, 32, 34) and motivation (items 8, 20, 21, 35, 40). The final score is the sum of the scores for each individual question, with a possible 160 points maximum. The maximum score for each scale was: self-confidence, 36 points; mental attitude and preparation, 32 points; stress control and anxiety, 44 points; concentration, 28 points; and motivation, 20 points. Questionnaire presents a high reliability (Cronbach's alpha index = 0.85), and explains the 63% of the total variance in soccer players [42]. In this study, the reliability of this test was medium-high (Cronbach's alpha index = 0.78). The motivation dimension was the lowest value and the stress control and anxiety dimension was the highest value for Cronbach's alpha index with 0.42 and 0.85, respectively. This dimension of motivation has not obtained a high value of reliability because there may be dispersion in responses among the group of futsal players evaluated.

### Design and procedures

The design of this study was descriptive and transversal. The study was done in November 2016 during the preliminary selections of players for the 2016/2017 season. At this point, pre-season is over and the first round of National League has begun. The study was conducted over two training practices.

At the first session, researchers explained to the players the aim and the actions of the study, and were given the voluntary consent form in order to sign their parents. During the second session, players brought signed the voluntary consent form; data on physical and social-demographic characteristics was collected and the Psychological Characteristics related to SP for soccer players Questionnaire was administered. The time interval between one session and another was 15 days that met the selection of players from different parts of Spain. The process was carried out respecting anonymity and confidentiality. Participants did not put their name on the questionnaires, and the questionnaires were not numbered until after all participants had completed them.

### Statistical analysis

Microsoft Excel 2010 (Microsoft Corp, Redmond, Washington, USA) and SPSS for Windows v.20.0 (IBM SPSS Statistic, Chicago, USA) were the software programs used. A Kolmogorov-Smirnov test was performed to check normality of the variables. Descriptive and comparative tests (t-test) were performed for the independent variables, category, family members present at matches, and experience (years federated). One-way analysis of variance test (ANOVA) was assessed on the five playing positions with Bonferroni post hoc in order to determine their differences. To evaluate the differences between various comparisons between groups, a multivariate general linear model (GLM) MANOVA was performed with playing position, experience and category as fixed factors. Lastly, the Pearson correlation coefficient was calculated to measure the relationship between different variables, and a linear regression analysis (stepwise) was used to find the independent variables which influence psychological states. Standardized units 0.20 (that is, a fraction of the standard deviation between participants at the beginning of this research) were selected as the least significant change [43]. The size of the effect (*η*^*2*^_*p*_) quantifies the size of the difference that exists between both groups; so, according to this, we could say that this is a true measure of the significance for such a difference [44]. The threshold values for the Cohen effect sizes [43] would be in t-test, small: 0.20; moderate, 0.50; and large, 0.80; and in ANOVA test, small: 0.10; moderate, 0.25; and large, 0.40. Significance level established was set at *p* < 0.05.

## Results

Players of different ages showed similar scores and percentiles of psychological characteristics. No significant differences were found between categories, although the U16 players indicated higher values in stress control, concentration, and motivation, while the U19 players showed higher values in self-confidence and mental attitude and preparation (*p* > 0.05). For this reason, these data informed that homogeneity between young promises futsal players had scarce variation.

Table 1 shows the scores and percentiles (mean and standard deviation) for psychological characteristics according to the playing position. The goalkeeper had higher scores in self-confidence and stress control (in points as well as in percentiles) in comparison to the pivots and wing-pivots, and the universals (*F*_*4*,*166*_ = 3.39, *p* = 0.01, *p* value *post hoc* = 0.007; *F*_*4*,*166*_ = 2.10, *p* = 0.05, *p* value *post hoc* = 0.05; respectively). In other psychological characteristics as mental attitude and preparation, concentration, and motivation, there were not any differences among playing positions (*p* > 0.05).

**Table 1:** Psychological characteristics of futsal players according to playing position.

		G (*N* = 31)			D—DW (*N* = 35)			W—WD (*N* = 33)			P—WP (*N* = 40)			U (*N* = 26)			*F*_*(4*,*166)*_	*p*	*η*^*2*^_*p*_	*Qualitative assessment*
SC	(points)	31.36	±	11.4^**Pi**^	25.67	±	3.18	26.50	±	4.08	22.32	±	7.55^**Go**^	23.00	±	3.16	3.39	0.01	0.23	Moderate
MA	(points)	21.45	±	6.38	22.00	±	4.75	22.25	±	4.88	23.95	±	4.63	22.40	±	4.28	0.61	0.65	0.10	Small
CS	(points)	34.27	±	8.46^**U**^	32.27	±	7.83	30.83	±	6.62	28.23	±	7.97	24.00	±	10.0^**Go**^	2.08	0.05	0.18	Moderate
CO	(points)	21.82	±	5.06	23.80	±	4.02	22.08	±	4.83	20.73	±	5.74	20.40	±	4.83	0.94	0.44	0.12	Moderate
MO	(points)	12.09	±	3.99	14.00	±	3.09	12.83	±	4.32	13.64	±	2.92	14.00	±	4.30	0.62	0.65	0.10	Small
Ʃ	(points)	121.0	±	21.8	117.7	±	14.8	114.5	±	17.0	108.9	±	19.5	103.8	±	16.8	1.37	0.25	0.15	Moderate
SC	(%)	87.09	±	31.4^**Pi**^	71.27	±	8.78	73.67	±	11.4	62.00	±	21.1^**Go**^	64.00	±	8.86	3.37	0.01	0.23	Moderate
MA	(%)	67.00	±	20.0	68.80	±	14.9	69.58	±	15.2	74.91	±	14.6	70.00	±	13.6	0.62	0.65	0.10	Small
CS	(%)	77.91	±	19.2^**U**^	73.40	±	17.8	70.25	±	15.1	64.14	±	18.1	54.60	±	22.7^**Go**^	2.10	0.05	0.18	Moderate
CO	(%)	77.82	±	18.1	85.00	±	14.4	78.83	±	17.2	74.00	±	20.5	73.00	±	17.3	0.94	0.44	0.12	Moderate
MO	(%)	60.45	±	19.9	70.00	±	15.5	64.17	±	21.6	68.18	±	14.6	70.00	±	21.5	0.62	0.65	0.10	Small

G: Goalkeepers D: Defenders; DW: Defenders-wings; W: Wings; WD: Wings-defenders; P: Pivots; WP: Wing-pivots; U: Universals; *η*^*2*^_*p*_: Effect size; SC: Self-confidence; MA: Mental attitude and preparation; CS: Control of stress; CO: Concentration; MO: Motivation; Ʃ: Summation; *Bonferroni post hoc*: Letter in exponential corresponds to the playing position.

**Fig 1:**
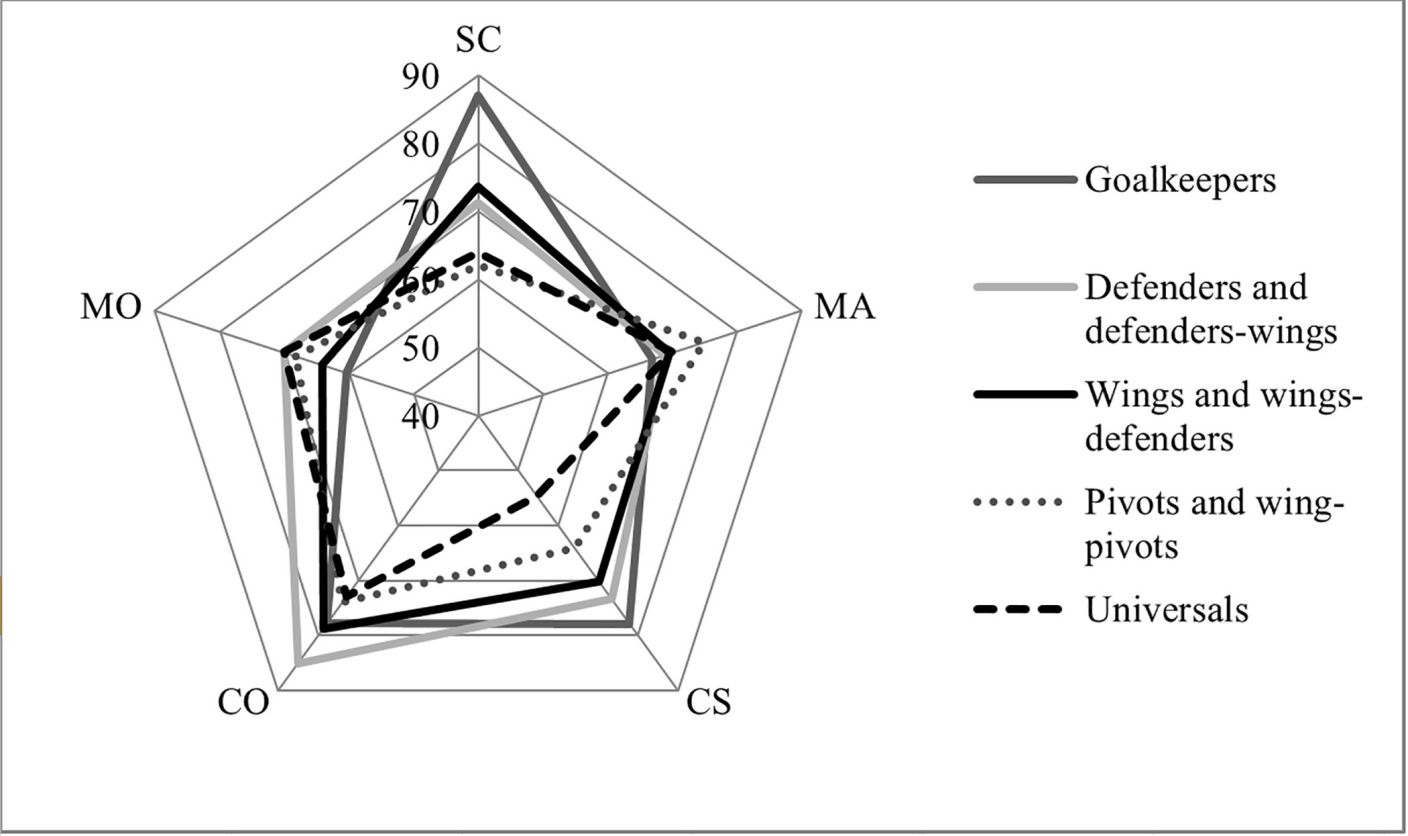
Psychological profile (%) according to playing position. SC: Self-confidence; MA: Mental attitude and preparation; CS: Control of stress; CO: Concentration; MO: Motivation.

Fig 1 shows the psychological profile of young promises futsal players as a function of their playing position. The prediction of stress control based on playing position and experience in years federated was tested by means of MANOVA. The results were a 14% of the explained variance (*F*_*1*,*166*_ = 1.88; *p* < 0.05; *η*^*2*^_*p*_ = 0.41; observed power = 0.78), although the interaction between playing position and experience was not significant. In this test, stress control is influenced by playing position (*F*_*4*,*166*_ = 2.59; *p* < 0.05; *η*^*2*^_*p*_ = 0.19; observed power = 0.68) and by experience (*F*_*1*,*166*_ = 4.48; *p* < 0.05; *η*^*2*^_*p*_ = 0.09; observed power = 0.54). The variable models explaining self-confidence were not significant, except for the playing position with a 20% of the explained variance (*F*_*1*,*166*_ = 3.37; *p* < 0.05; *η*^*2*^_*p*_ = 0.19; observed power = 0.82).

Lastly, correlations were found between experience and motivation and between experience and the summation of psychological characteristics related to performance (*r* = 0.40 and 0.33; *p* < 0.01; respectively), as well as between playing position and, in turn, self-confidence, stress control and the summation of psychological characteristics (*r* = - 0.38; - 0.34; - 0.29; *p* < 0.01; respectively; Table 2). These last negative correlations mean that the higher playing position, lower scores obtained in the psychological characteristics, and vice versa. The category of playing position was identified as ordinal, with the goalkeeper being the value 1 and the pivot and wing-pivot as value 5.

**Table 2:** Correlations between psychological characteristics and experience and playing position in futsal.

	Experience	Playing position
SC	-0.197**	-0.382**
MA	-0.066	0.112
CS	0.041**	-0.342**
CO	0.193**	-0.168**
MO	0.397**	0.109**
Ʃ	0.334**	-0.287*

* p < 0.05

** p < 0.05; SC: Self-confidence; MA: Mental attitude and preparation; CS: Control of stress; CO: Concentration; MO: Motivation; Ʃ: Summation

## Discussion

The aim of this study was to identify the psychological characteristics related to SP and the psychological profile of young promises (U16 and U19) futsal players among to the playing position. Generally, all coaches and physical trainers look for information about the variables and factors that could affect the performance of their players. This interest is even greater when higher levels of play are involved, resulting in the need to know manage physiological, anthropometric, nutritional, physical, technical, tactical, and psychological factors [26] in order to make decisions for the benefit of the team. This need is shared by Palucci et al. [37], who encourage investigators to increase the number of studies that could help to improve the SP.

The psychological characteristics related to SP, found in U16 and U19 players, were similar. This similarity of characteristics between categories and/or age has been found in other comparable sports, such as soccer [45]. This commonality may be attributed to the homogeneity of playing level among the players, given that all were young promises futsal players who had been selected for the Autonomous Community team. So, it could be concluded that psychological responses is important for young promises futsal players, but that homogeneity between players results with a little variation.

Nevertheless, in our study, futsal players showed different psychological characteristics among to playing position, regardless of the category. These playing positions in the playing field were categorized according to defensive or offensive roles, resulting in goalkeepers, defenders and defenders-wings, wings and wings-defenders, pivots and wing-pivots, and universals. This categorization is a revision and an adaptation of the initial classification proposed by Santana [38] and Caetano et al. [46]. This new classification collects the current movement of players. Today, players have a more dynamic role than in the past when the covered player area was limited.

Goalkeeper players showed greater self-confidence and stress control than the rest of positions, the pivots and wing-pivots, the least self-confidence, and the universals had the lowest scores in stress control. These findings are important as they confirm results found in research studies as well as in the professional field. For example, in handball sport, which has the same playing pitch and other similar characteristics, Olmedilla et al. [4] concluded that the goalkeepers had greater stress control, and in general, a better profile for greater SP than other positions; and Paz-Franco et al. [35] and Mutti [34] determine that these players have excellent conditions in order to compete.

In futsal, higher scores in the summation of psychological characteristics (self-confidence, mental attitude and preparation, stress control and anxiety, concentration, and motivation) were found in the goalkeepers, too. These scores diminished as the playing position became more offensive. In other studies of futsal where the psychological characteristic is limited to stress control, different correlations were found. Soares-Caldeira et al. [10] found a reduction of stress and improved the SP in high-level futsal players who carried out sprints during training sessions. Milanez et al. [9], in a five-week interventional study of female players, observed a clear association between the training workload and the symptoms of stress. Specifically, a reduction of the stress symptoms corresponded to an increased training workload. Weinberg and Gould [23] and Lorenzo et al. [24] established a positive relationship between the age and experience and stress control of players. In another study, Geisler and Kerr [6] analyzed stress in futsal players in Canada and Japan one hour after competitions. Results showed that the Japanese players felt more stress after a defeat, while the Canadian players felt more stress after a victory.

In studies where the psychological characteristic studied was self-confidence, Pujals and Fiorese [17] found that low self-confidence is related to stress and anxiety. The studies of Gimeno et al. [18] and González et al. [19] were in agreement, demonstrating an inverse relationship between self-confidence and anxiety in soccer players. In fact, self-confidence facilitates better performance in many different sport situations [21, 22]. These situations are found in competition matches through observational methodology and allow the technical staff to check if an soccer player has a high number of errors [47, 48].

In our study, self-confidence dimension was predicted by the 20% of the explained variance for playing position factor. In this sense, there are no studies that have made this prediction between self-confidence and the playing position. However, Sepasi et al. [49] indicated that considering that up to 28% of variance in sport success can be explained by the variable self-confidence, there is a need in order to carry out intervention programs for trainers which help them to develop this and other similar psychological capacities in their players [5, 50]. Accordingly, it is vital that further research on futsal be done, with the objective of determining precisely all the factors that contribute to maximum SP. For future studies, Yeemin et al. [1] recommend a longitudinal design and a focus on the younger players, and furthermore, it is need to assess the psychological characteristics fluctuation during a sport season according to playing position.

On the other hand, no differences were found in the dimensions mental attitude and preparation, concentration and motivation, depending on the playing position. The concentration of the athlete is an attentional process that focuses all his mental effort on different tasks without being distracted, achieving sports success [51]. The defensive behavior, aimed mainly at the recovery of the ball [30], requires a high level of concentration [29] in order to make decisions and develop individual and collective behaviors that avoid unnecessary risks. For this reason, there is a high interest in studying these attentional processes in team sports and relating them to the motivation that this sport implies [52]. In the present study, motivation has been related to the futsal player's experience. A study showed that high values of mental toughness were associated with higher levels of experience of the athlete [53], so this had repercussions on the motivation of the athlete in the sport [2].

The main novelty of this study is that multiple psychological constructs (self-confidence, mental attitude and preparation, control of stress, concentration, and motivation) have been described in elite young players. It has been revealed that these psychological responses differ in a very homogeneous population, as the players selected by a region to play the National Championship Selections. These differences, which are related to personality, have been shown according to their playing position, category and experience. For these reasons, this study is presented as a pioneer in this line because it indicates that players with different roles on the field have different psychological responses. Although the results of this study are of special interest from a psychological point of view to help the technical staff know the personality of the athlete, this study also presents some limitations. Firstly, knowing the psychological profile of elite young athletes makes it difficult for teams to give permission to investigate these psychological aspects. Therefore, a larger sample may allow more scientifically rigorous conclusions to be established. However, this sample may represent 10% of the elite futsal players of developmental age, which is a strong point of the study. A positive aspect could be the inclusion of the level of expertise to check whether the psychological profile differs between players with higher and lower level of play. On the other hand, a longitudinal study can allow knowing the fluctuation of the futsal players during the season [2], which would extend the real information of the psychological profile. Finally, to establish differences between the moments before the competition with the basal states can contribute the difficulty of the futsal players when facing the competition factor.

## Conclusions

The main finding of this study contributes to growing of scientific literature on the futsal topic, showing that young promises futsal players in more offensive positions (e.g., the pivots, wing-pivots, and universals) have lower scores of the psychological characteristics, as stress control, and concentration than other positions. Therefore, these findings confirm the starting hypothesis that goalkeeper position presents the best psychological characteristics, and profile for SP. These conclusions should serve the coach so that such characteristics will constitute the individualities, as well as the ways of training and working psychologically with each player. In addition, they should be taken with caution because the sample is of young promises players, so these, and we urge other researchers to further examine the effect of the offensive or defensive role on the psychological variables in other categories of players as sample.

## Supporting information

S1 Data Minimal data set. (SAV)
